# Clinical Outcomes and Patient Safety of Nasogastric Tube in Acute Stroke Patients

**DOI:** 10.1007/s00455-022-10437-1

**Published:** 2022-03-16

**Authors:** Jodie Rabaut, Tharani Thirugnanachandran, Shaloo Singhal, Julie Martin, Svitlana Iievliev, Henry Ma, Thanh G. Phan

**Affiliations:** 1 Stroke Unit, Monash Health, Clayton, Australia; 2 Department of Neurology, Monash Health, 246 Clayton Road, Clayton, Australia; 3 Clinical Trials, Imaging and Informatics (CTI) Division, Stroke & Ageing Research (STAR), Department of Medicine, School of Clinical Sciences at Monash Health, Monash University, Clayton, VIC 3168 Australia

**Keywords:** Stroke, Dysphagia, Nutrition, Nasogastric tube, Pneumonia

## Abstract

Nasogastric tube (NGT) is often used in stroke patients who are dysphagic (deglutition disorders) or have decreased conscious state. This method of feeding is assumed to have minimal complications. The aim of this study is to analyze complications associated with NGT and variables associated with mortality. Retrospective analysis of 250 acute stroke patients requiring NGT feeding between 2003 and 2020. There were 250 patients (median age 76 (IQR 68–83), 56.4% males, median time to NGT 1 day (IQR 0–3). Discussion with family prior to insertion of NGT recorded in 46 (18.4%). There were 123 cases (49.2%) of aspiration pneumonia. There were 188 (75.2%) NGT associated complications: 67 patients (26.8%) had failed insertion, 31 required multiple attempts, 129 patients (51.6%) pulled out NGT, 107 patients (42.8%) had NGT placed in wrong positions and require reinsertion, 20 cases in the lung, 5 pneumothorax cases, 97 in the gastro oesophageal junction or hiatus hernias, 1 case of oesophageal ulceration, 37 coiled, kinked or resistance. 78 cases the tips were not seen on chest X-ray (CXR), gastrointestinal bleeding in 9 cases, epistaxis in 6 cases), 96 patients (38.4%) required restrain. There were 91 death (36.4%) with 73 patients occurring during hospital admission and a further 18 died within 6 months. Death was more frequent in those age > 60 (72 of 216 patients versus 1 of 33 patients, *p* < 0.01). The median National Institute of Health Stroke Score/NIHSS of those with aspiration pneumonia was higher than those without (19.5 versus 15, *p* < 0.01). Decision tree analysis first split at age (≤ 59 versus > 59, *p* = 0.03), NIHSS (≤ 16 or > 16, *p* = 0.02), post-stroke pneumonia (*p* = 0.04) and multiple NGT insertion (*p* = 0.01). The area under the ROC curve was for this model was 0.75 (95% CI 0.69–0.80). Complications were common among patients with NGT complications. These findings may be used to inform discussions with families regarding NGT.

## Introduction

Stroke is a leading cause of disability worldwide and results in significant economic and societal cost [1]. The Feed Or Ordinary Diet (FOOD) trial has raised awareness of the importance of nutrition in stroke outcome [2]. The concern with poor nutritional status affecting the outcome of stroke patients has led to the consideration of nasogastric tube (NGT) and percutaneous endoscopic gastrostomy (PEG) feeding among severely dysphagic (deglutition disorders) patients [2, 3]. The question on when to use PEG feeding versus NGT had led the FOOD investigators to perform that randomized trial [4]. The result of the FOOD trial was difficult to understand as the investigators reported that “Early tube feeding might reduce case fatality, but at the expense of increasing the proportion surviving with poor outcome” [5]. Importantly the FOOD investigators reported 2- to threefold increased risk of gastrointestinal bleeding among patients receiving NGT. Other complications of NGT insertion such as pneumothoraxes were not mentioned in the FOOD trial nor commented upon in guidelines as potential complications [6]. Guidelines often suggest the use of NGT to deliver antiplatelet therapy among patients who are kept nil by mouth (NBM) [4, 7]. More recently, the use of NGT has been proposed in the Australian Living Guidelines as a mean to deliver paracetamol to treat fever in patients with dysphagia [4]. The aim of this study is to analyze complications associated with NGT and determine variables associated with mortality. This data will be useful for informed consent in discussion with patients, their carers and relatives.

## Method

This is a retrospective study of admissions to the stroke unit between 2003 and 2020. Patients were identified by search for nasogastric tube in the discharge summary and handover sheet. We collected data on demographic variables, admission diagnoses, time to triage, imaging, screening for dysphagia, nil by mouth (NBM) status, stroke severity (National Institute of Health Stroke Scale or NIHSS). The NIHSS has values ranging from 0 (no signs) to 42 (intubated patient). Stroke is defined as minor stroke if NIHSS ≤ 4, moderate for NIHSS between 5 to 15, and severe for NIHSS > 15. Stroke is defined as severe. In this study, the NIHSS was taken from the clinical examination performed on admission. The diagnosis of post-stroke pneumonia was made by the treating team. It was defined clinically by the finding of fever (temperature ≥ 38 °C), consolidation on chest radiograph, and use of antibiotic medications [8]. This study was approved by Human Research Ethics Committee. A waiver of individual consent was granted given that the study was retrospective in nature and there was no intervention component to this study.

### Swallowing Assessment

Suspected stroke or confirmed stroke patients are required as per hospital procedure to remain NBM until a dysphagia screen has been performed. This procedure can be performed by a trained nurse or by a speech pathologist. The dysphagia screen tool used was Acute Screening of Swallow in Stroke or TIA (ASSIST) tool [9]. Patients who passed the dysphagia screen were permitted to have oral intake, while those patients failing the screen remained NBM until reviewed by a speech pathologist within 24 h of admission. The speech pathologist then either allowed the patients to have a modified diet or required that they remain NBM or have nasogastric tube insertion. Patients were kept NBM if they were drowsy, unable to swallow their saliva, have sign of aspiration or have significant bulbar weakness on assessment. Practices have changed over the years. Currently patients who remained NBM are also assessed by dieticians and the decision is made on day 3 regarding the need for NGT.

#### NGT

The procedure for performing NGT is as followed: check nasal passages to ensure no obstruction. Measure the length of the tube (silicone fine bore NGT's Covidien Kangaroo 12FR/CH 4.00 × 109 cm) by placing the exit port at the tip of the nose, extend to the earlobe then to the xiphoid process. Tube measurement is used to estimate the length required to place the NGT into the stomach. The tip of NGT is lubricated and inserted into the nasal flare, follow the roof of the nostril into the pharynx. The patient is asked to tilt the head forward to open the esophagus, narrowing entry to the trachea. Insertion continues until the right length of the NGT measurement has been reached. The oral cavity is checked to ensure that the tube is not coiled in the mouth or back of throat. The NGT is secured with nasofix adhesive tape and the patient is sent for chest X-ray (CXR) to confirm placement.

Conditional decision tree analysis was used to find demographic variables associated mortality performed within R programming environment. The tree construction was performed using the *party* package in R [10]. Classification of a binary response variable can be viewed as having a set of rules that are applied sequentially, with each rule partitioning an attribute (predictor variable) into a binary response. This approach has theoretical advantage over the CART method for decision tree because that method uses information criterion for partitioning and which can lead to overfitting [11]. The area under ROC curves were compared using *pROC* package in R.

## Results

There were 250 patients [median age 76 (IQR 68–83), 56.4% males]. The median NIHSS was 17 (IQR 10, 23). The breakdown of the NIHSS and frequency of pneumonia according to the patients’ discharge disposition is presented in Table 1.

**Table 1:** Patient characteristics

	Minor stroke	Moderate stroke	Severe stroke	Pneumonia	Position complications	Insert complications	Total
Home	3 (42.9%)	1 (14.3%)	3 (42.9%)	0 (0%)	2 (28.6%)	0 (0%)	7
Rehabilitation	19 (17.3%)	42 (38.2%)	49 (4.5%)	47 (42.7%)	50 (45.5%)	31 (28.2%)	110
GEM	1 (4.0%)	8 (32%)	16 (64.0%)	9 (40.0%)	15 (60%)	13 (52%)	25
Nursing Home	1 (3.3%)	8 (26.7%)	21 (70.0%)	20 (66.7%)	13 (43.3%)	10 (33.3%)	30
Died	3 (4.1%)	19 (26.0%)	51 (69.9%)	44 (60.3%)	30 (41.1%)	18 (24.7%)	73

*GEM* Geriatric Evaluation and Management unit

**Fig. 1:**
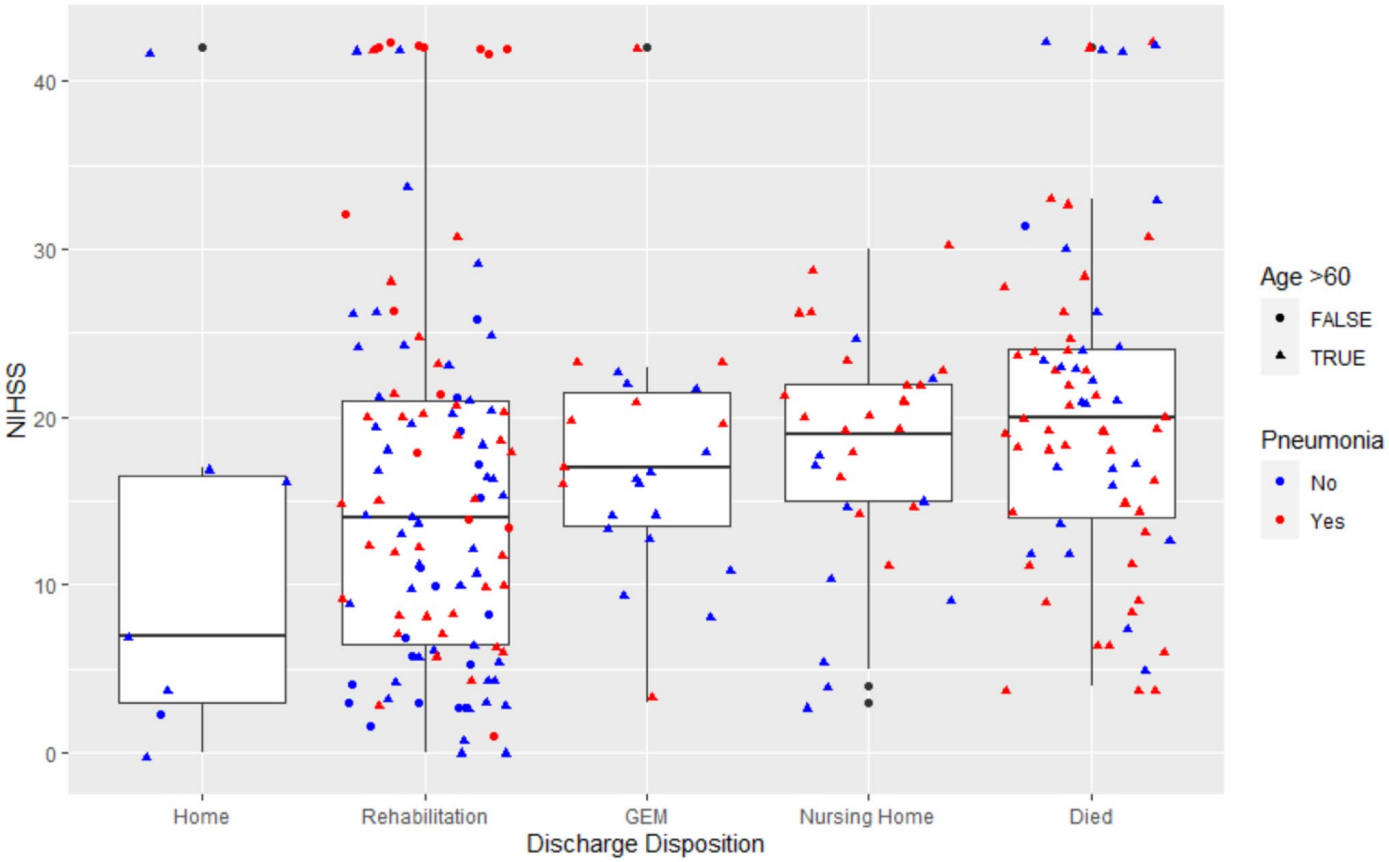
Discharge disposition versus stroke severity. The figure shows the relationship discharge disposition and stroke severity (NIHSS). Patients who were discharged home had mild stroke while those who died or were looked after in Geriatric Evaluation and Management (GEM or slow stream rehabilitation) and nursing home also had high stroke severity. Patients who died (represented by circles) are 60-year or older, have pneumonia (red color) and severe stroke. After adjusting for Bonferroni correction, there was significant difference in NIHSS between Rehabilitation group and those who died (*p* = 0.018). There was no statistically significant difference between the other groups in terms of NIHSS

**Fig. 2:**
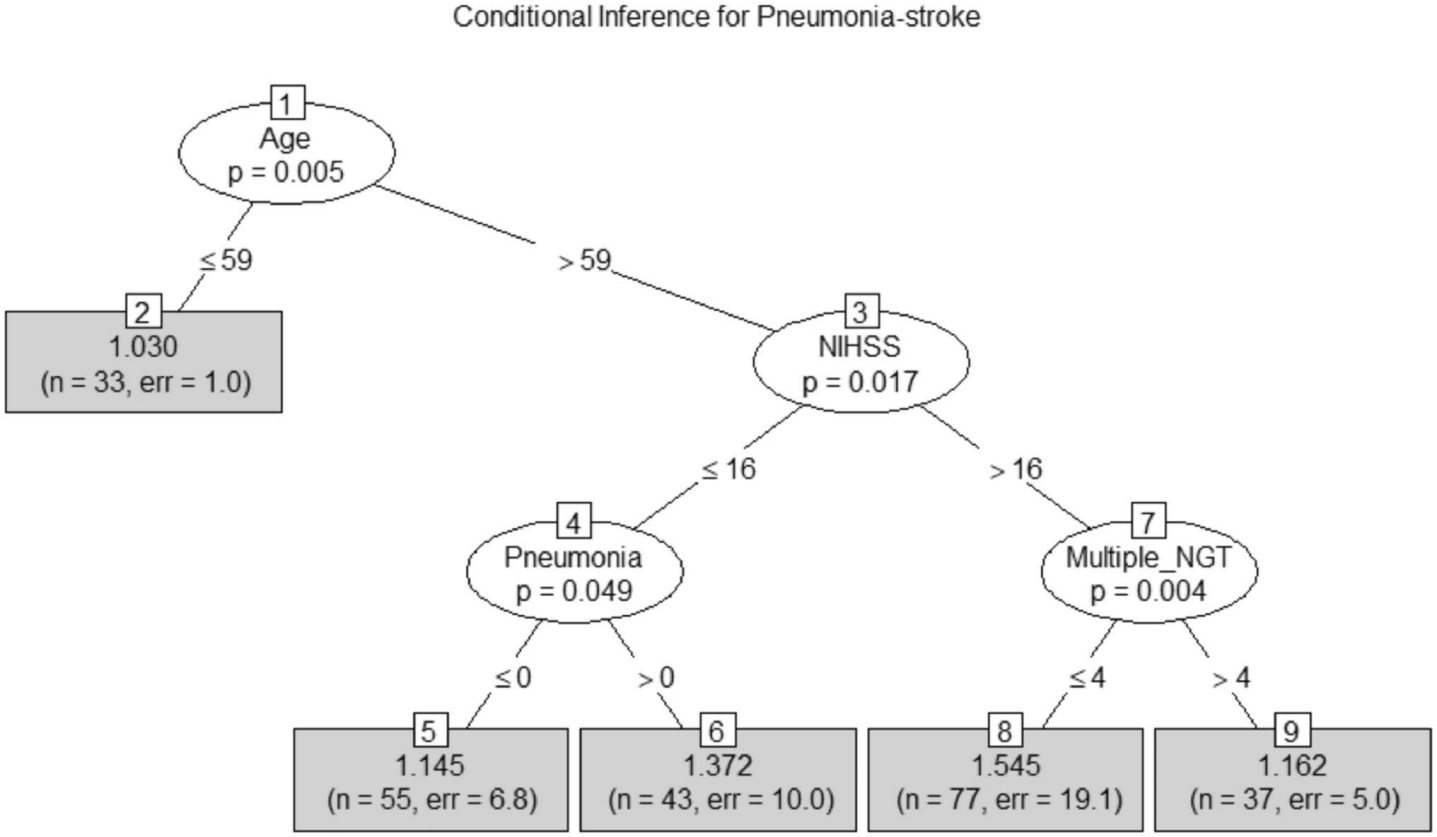
Decision tree model of mortality. The decision tree shows the hierarchy of variables associated with mortality. Age and stroke severity (NIHSS) dominate the outcome of mortality. Patients with pneumonia and NIHSS lower than 16 are more likely to die. Patients having multiple nasogastric tube (NGT) insertion. The area under the ROC curve was 0.75 (95% CI 0.69–0.80)

The prevalence of hypertension was 195 (78%) cases, ischemic heart disease 65 (26%) cases, diabetes 76 (30.4% cases, hyperlipidemia 89 (35.6%), ever smoker 57 (22.8%) cases. Stroke involved exclusively anterior circulation in 184 cases, posterior in 42 cases, and combined territories in 14 cases. There were 112 (44.8%) cases discharged to rehabilitation, 31 (12.4%) cases discharged to nursing home, 25 (10%) cases discharged to Geriatric Evaluation and Management (GEM) or slow stream rehabilitation, 7 (0.28%) cases discharged home (Fig. 1). The median time to NGT 1 day (IQR 0, 3). The time from admission to resumption of oral intake was 3 days (IQR 1, 8) while the time from NGT to resumption of oral intake was 2 days (IQR 1, 8). The median time from commencement of oral intake to removing of NGT was 1 day (IQR 0, 6). In 101 cases, NGT was inserted after commencement of oral intake and supplement nutrition. There were 123 cases (49.2%) of aspiration pneumonia (Fig. 2). There were 188 (75.2%) NGT associated complications: 67 patients (26.8%) had failed insertion, 31 required multiple attempts, 129 patients (51.6%) pulled out NGT, 107 patients (42.8%) had NGT placed in wrong positions and require reinsertion 20 cases in the lung, 5 cases of pneumothoraxes, 97 in gastro oesophageal junction or hiatus hernias, 1 case of oesophageal ulceration, 37 coiled, kinked or resistance. 78 cases the tips were not seen on CXR, gastrointestinal bleeding in 9 cases, epistaxis in 6 cases, 96 patients (38.4%) required restraints. Discussion with family prior to insertion of NGT recorded in 46 (18.4%). Discussions with the family regarding risk with the procedure was recorded in 19 (7.6%). There were 91 death (36.4%) with 73 deaths occurring during hospital admission, and a further 18 died within 6 months. Death was more frequent in those age > 60 (72 of 216 patients versus 1 of 33 patients, *p* < 0.01). The median NIHSS of those with pneumonia was higher than those without (19.5 versus 15, *p* < 0.01).

### Decision Tree Analysis of Mortality

**Fig. 3:**
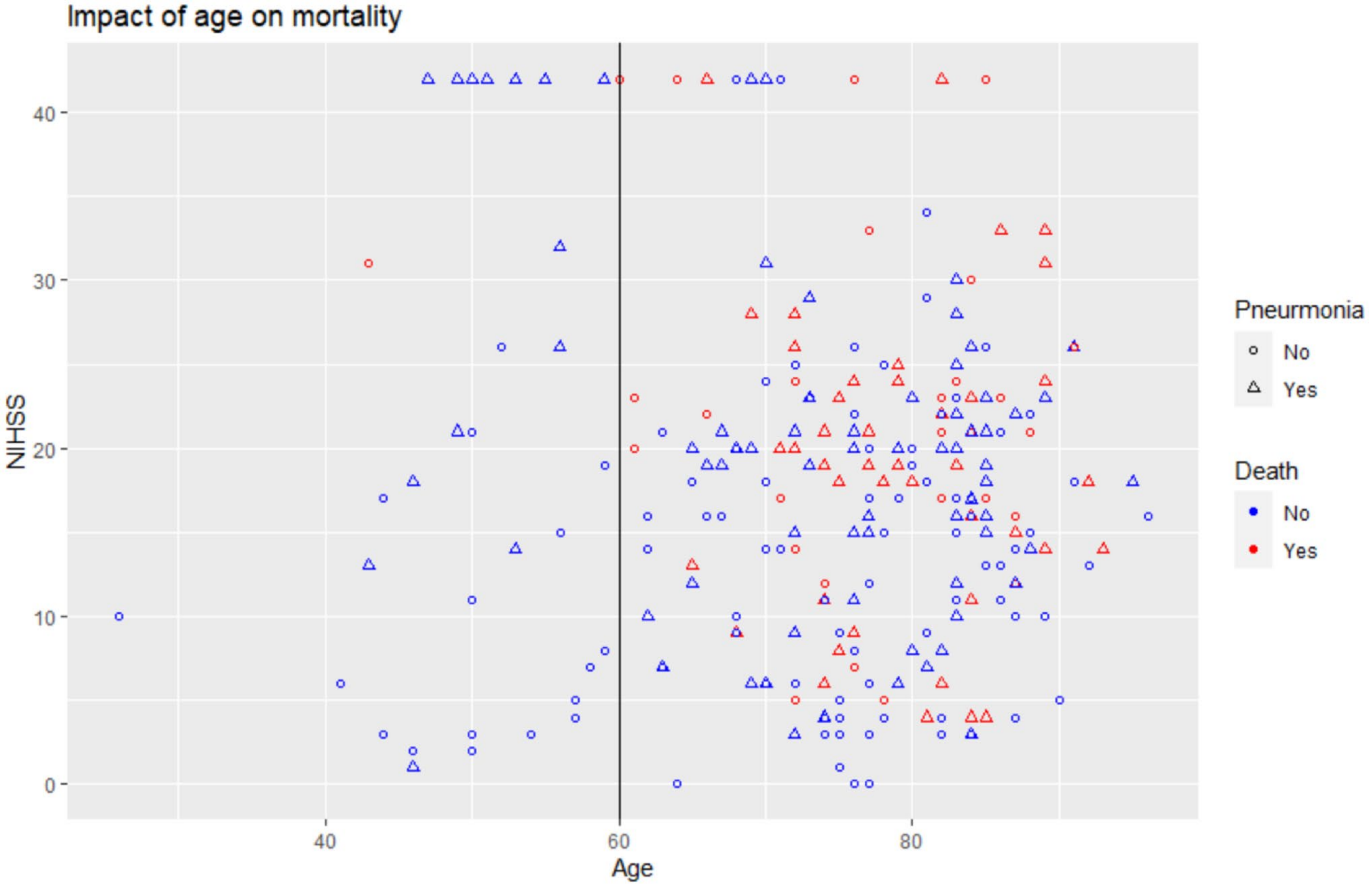
Age, stroke severity, and multiple nasogastric tube (NGT) insertion. This figure reflects the findings of the decision tree. It shows the relationship between higher mortality (red color) among older patients

**Fig. 4:**
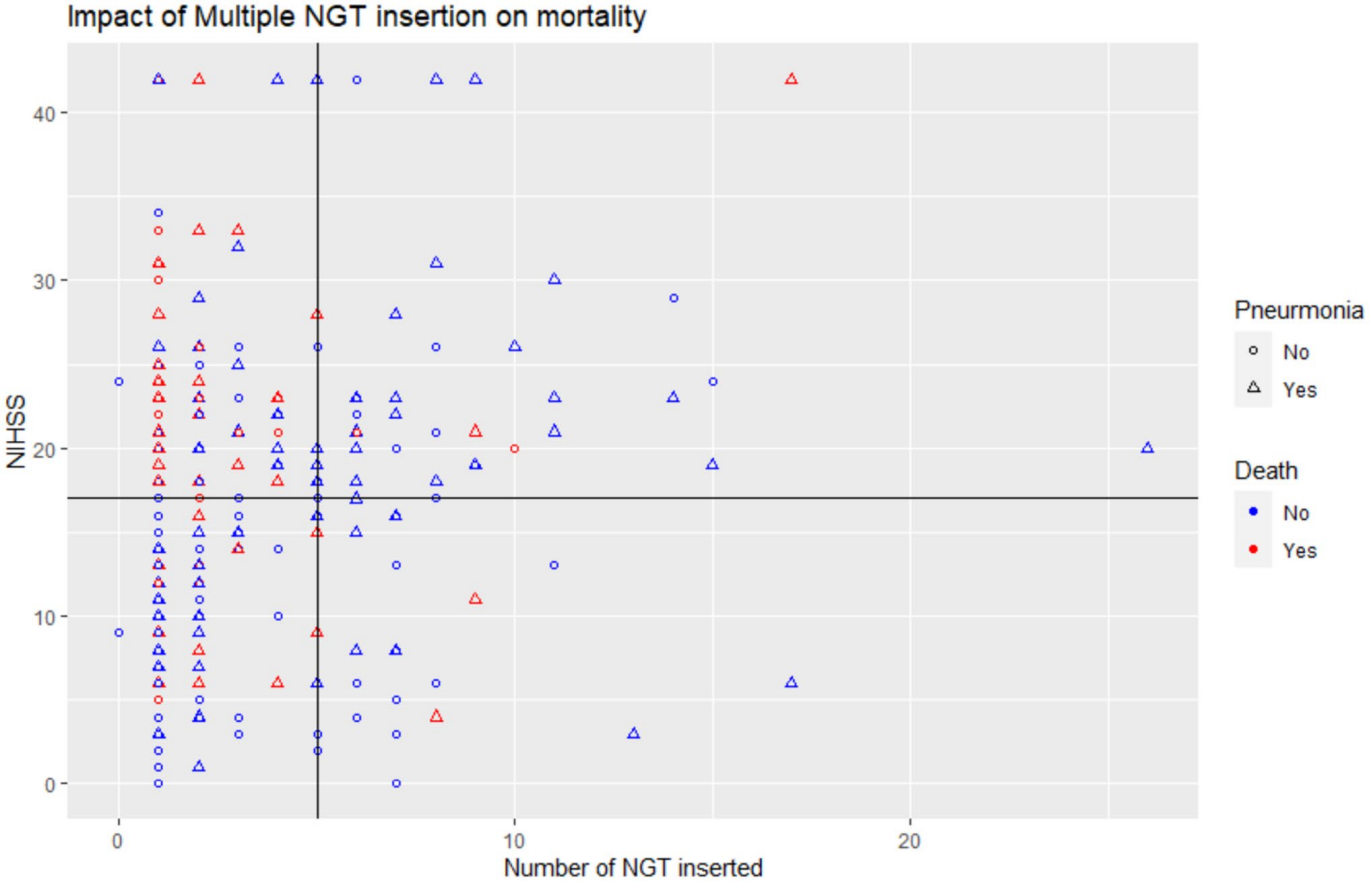
Impact of Multiple NGT insertion on mortality. This figure shows that patients with multiple nasogastric tube (NGT) insertion (threshold at greater than 4, see vertical line) were less likely to die

The decision tree was limited to 245 patients due to missing NIHSS in 5 patients. Decision tree analysis first split at age (≤ 59 with 33 patients versus > 59 with 212 patients, *p* = 0.03, Fig. 2). Only one patient died among those less than 60-year-old. Among 212 patients with age (> 59), the tree is split again at NIHSS (≤ 16 with 88 patients or > 16 with 124 patients, *p* = 0.02). Among 88 patients with NIHSS ≤ 16, 43 patients had pneumonia and 16 died. Among the 55 patients who did not have pneumonia, 8 died. The presence of pneumonia conferred high probability of death (*p* = 0.04). Among 114 patients with NIHSS > 16, multiple NGT insertion (6 of 37 with multiple NGT insertion died versus 42 of 77 with smaller number of NGT insertion died (*p* = 0.01). The area under the ROC curve (auROC) was for this model was 0.75 (95% CI 0.69–0.80). To better explain the findings of the decision tree, we have created further plots shown in Figs. 3 and 4. Figure 3 shows the relationship between age and mortality. Figure 4 shows the relationship between NIHSS and lower number of NGT inserted.

### Logistic Regression

The logistic regression shows that the multivariable model was significant for Age (OR = 1.008, 95% CI 1.003–1.012, *p* = 0.0009), NIHSS (OR = 1.008, 95% CI 1.002–1.013, *p* = 0.005), and multiple NGT insertion (OR = 0.968, 95% CI 0.951–0.986, *p* = 0.0006).

## Discussion

In this cohort, complications were common among patients having NGT insertion. Our findings must be put in context as these patients have severe stroke (NIHSS) and impaired ability to cooperate with NGT insertion compared to other patients in the hospital such as surgical patients. The second part of the analysis concerns prediction of outcome of mortality. This analysis showed that older age, severe stroke, pneumonia, and number of nasogastric tube insertion were associated with mortality. These findings may be used to inform discussions with families of stroke patients regarding NGT.

### Complications

We had observed a range of complications of NGT ranging from incorrect placement to gastrointestinal hemorrhage. The complications of NGT are known but are rarely discussed [12]; pharyngeal misplacement of NGT was noted in 5 of 100 cases [12]. The FOOD investigators had commented on the higher frequency of hemorrhage but did not comment on other known complications such as pneumothoraxes [6]. Similarly, a comparison of NGT and PEG using matched design did not discuss the chest complications [13]. A systematic review on the topic noted that only 6 of 12 papers on the topic described NGT placement failure and malposition [14]. The authors concluded “Data are scarce on the topic. Research about the frequency of local and systemic NGT complications and strategies for prevention will certainly contribute to enhance evidence-based management of dysphagia in acute stroke” [14]. Search for specific terms relating to chest complications were not found in the papers reviewed in this meta-analysis [14]. By contrast, NGT insertion was better documented outside of the stroke literature [15–19]. In one study, investigators described 50 cases of malposition with small bore NGT over 4 years period [15]. Importantly, half of these patients were mechanically ventilated. Only 2 of these patients had ‘normal’ mental status according to the authors [15]. In another study, the authors reviewed complications of NGT placement and noted even rare instance of NGT entering the brain [16]. It is possible that the documentations of NGT misplacement was high in our data because of the requirement for CXR to be performed in all patients. Alternative approach includes the use of smart sensor guided nasogastric tubes (enteral access system). However, there are warnings on these enteral access system regarding reports of 51 pneumothoraxes and 11 death over 5 year period [20].

In our study, documentation of prior discussions with the family was poor. This could have been due to poor documentation in general or due to perception that the procedure had low risk [21]. A review of the training manual developed by the State of Victoria suggested that consent is required prior to nasogastric tube insertion [22].

### Pneumonia

The rate of pneumonia among our patients with NGT is high and is consistent with observations in the literature [5, 23]. One study reported rate of 44% while another reported rate of 76% by day 7 [5, 23]. Our finding on the importance of stroke severity to post-stroke pneumonia is consistent with that observed by Australian investigators made similar finding who used immobility as surrogate for stroke severity [24]. There is conflicting evidence on the use of metoclopramide in preventing post-stroke pneumonia among patients who had NGT feeding [25, 26]. One randomized trial suggested that metoclopramide delayed onset of pneumonia but did not affect development of pneumonia. In 2021, the European Stroke Organization Guidelines suggest using metoclopramide to “promote gastric emptying and reduce the risk of esophago-pharyngeal regurgitation with subsequent aspiration” [21]. The Guidelines did acknowledged that this recommendation was based on low quality evidence and the strength of recommendation was weak [21].

### Mortality

Using a decision tree approach, we construct a hierarchy of variables associated with mortality among patients with nasogastric tube. In our cohort, those age 60 or more with high stroke severity were more likely to die after NGT insertion. This was the case even among those above age of 60 and NIHSS below 10. The findings are of use when discussing with patient and family on prognosis.

### Proposal for Improvement

The lessons from this audit will be used to improve our nasogastric tube insertion, namely the use of consent and data from this project to inform patients and carers. An understanding of the potential complications can aid in the process of insertion. One group proposed this strategy to reduce complications: “adoption of a more compliant feeding tube, direct supervision of residents, technology-guided insertion, and implementation of explicit policies and procedures” [11]. We can add inserting nasogastric tube in the morning by an experience operator. This will give time to perform CXR to evaluate for potential complications. A variety of interventions have been proposed to aid maintenance of NGT in place such as mittens and nasal bridle, but these are not supported by evidence [27].

### Limitations

The data provided here comes from one institution. One issue with obtaining retrospective data is that it is not always possible to obtain time stamped data. As such we were unable to document the timing of the procedure. Due to the lack of documentation of the procedure, we were not able to infer the experience of the person performing NGT insertion. It is possible that we have not been able to identify all cases as we had based our approach on discharge summary and handover notes. Another potential issue is our approach to documenting complications in which we had included cases of incorrect placement to requirement for restraint to prevent self-removal of NGT. Incorrect placement has the potential for The National Health Service (NHS) had issued an alert safety number NHS/PSA/RE/2016/006 regarding 95 cases of fluid or medications had entered the lung from misplaced tube [28]. The alert was designed to alert medical staff to check placement of the tube. The ICD-10 code for complications from NGT is T85.528. However, this code describes any complication and is not billable.

## Conclusion

Complications of nasogastric tube insertion are common in stroke patients. Future publications on the subject should focus on complications in addition to their role in feeding stroke patients with severe dysphagia.
